# Residue K28 of Zika Virus NS5 Protein Is Implicated in Virus Replication and Antagonism of STAT2

**DOI:** 10.3390/microorganisms12040660

**Published:** 2024-03-26

**Authors:** Nias Y. G. Peng, Julian D. J. Sng, Yin Xiang Setoh, Alexander A. Khromykh

**Affiliations:** 1 School of Chemistry and Molecular Biosciences, The University of Queensland, St. Lucia, QLD 4072, Australia; j.sngdejun@uq.edu.au (J.D.J.S.); jsetoh@gmail.com (Y.X.S.); 2 Infectious Diseases Translational Research Programme, Yong Loo Lin School of Medicine, National University of Singapore, Singapore 119077, Singapore; 3 Australian Infectious Diseases Research Centre, Global Virus Network Centre of Excellence, Brisbane, QLD 4072, Australia

**Keywords:** flavivirus, Zika virus, innate immunity, virus–cell interaction, mutagenesis, replication, NS5, STAT2

## Abstract

The identification of four potential nonstructural 5 (NS5) residues—K28, K45, V335, and S749—that share the same amino acid preference in STAT2-interacting flaviviruses [Dengue virus (DENV) and Zika virus (ZIKV)], but not in STAT2-non-interacting flaviviruses [West Nile virus (WNV) and/or Yellow fever virus (YFV)] from an alignment of multiple flavivirus NS5 sequences, implied a possible association with the efficiency of ZIKV to antagonize the human signal transducer and activator of transcription factor 2 (STAT2). Through site-directed mutagenesis and reverse genetics, mutational impacts of these residues on ZIKV growth in vitro and STAT2 antagonism were assessed using virus growth kinetics assays and STAT2 immunoblotting. The results showed that mutations at the residue K28 significantly reduced the efficiency of ZIKV to antagonize STAT2. Further investigation involving residue K28 demonstrated its additional effects on the phenotypes of ZIKV-NS5 nuclear bodies. These findings demonstrate that K28, identified from sequence alignment, is an important determinant of replication and STAT2 antagonism by ZIKV.

## 1. Introduction

Although nonstructural 5 (NS5) protein sequences are highly conserved between many clinically important mosquito-transmitted flaviviruses, such as Dengue virus (DENV), Zika virus (ZIKV), West Nile virus (WNV), and Yellow fever virus (YFV), several differences exist between respective NS5 proteins, especially in the modulation of the host’s innate immune responses [1,2,3,4,5,6,7,8]. A prime example would be the antagonism of the type I interferon (IFN-I; IFNα/β) signaling pathway. The IFNα/β signaling pathway begins with the binding of secreted IFNα/β to two receptor subunits, IFNα/β receptors 1 and 2 (IFNAR1 and IFNAR2), with this interaction triggering the phosphorylation of associated Janus kinases (JAK), Tyk2, and JAK1 [9,10]. This initiates a transcriptional cascade involving the phosphorylation and heterodimerization of signal transducers and activators of transcription factors 1 and 2 (STAT1 and STAT2) [11,12]. The STAT1-STAT2 dimers then interact with IFN regulatory factor 9 (IRF9) to form the interferon-stimulated gene factor 3 (ISGF3) complex, which then translocates to the nucleus and binds to IFN-stimulated response elements (ISREs) to induce the expression of antiviral interferon-stimulated genes (ISGs) [13,14,15].

Among the clinically important flaviviruses mentioned earlier, only DENV and ZIKV are shown to antagonize STAT2 signaling activity by facilitating proteasomal degradation of STAT2 via direct NS5 interaction in the cytosol or the nucleus [1,3,6,7,16]. Furthermore, ZIKV can also antagonize STAT2 activity by directly interacting with STAT2 to prevent its binding to IRF9 and the formation of the ISGF3 complex [17]. Instead, other flaviviruses, such as WNV, inhibit the phosphorylation of STAT1 [4] or, like YFV, conditionally interact with STAT2 only in the presence of phosphorylated STAT1 to subvert ISGF3 signaling and the ISRE-mediated expression of ISGs [18].

Previously, Dar et al. performed an in silico study which predicted several ZIKV-NS5 surface residues that physically interact with human STAT2 [19]. Since then, several residues in ZIKV-NS5 protein have been demonstrated to be involved in the antagonism of STAT2 via a structural approach by Wang et al. [17]. However, not all of the NS5 residues predicted by Dar et al. [19] and Wang et al. [17] were experimentally validated at the time of our study’s commencement. Here, we report the experimental findings on four of the predicted NS5 residues and their roles in virus replication and STAT2 antagonism.

## 2. Materials and Methods

### 2.1. Sequence Alignment and Analysis of NS5 Sequences

**Figure 1:**
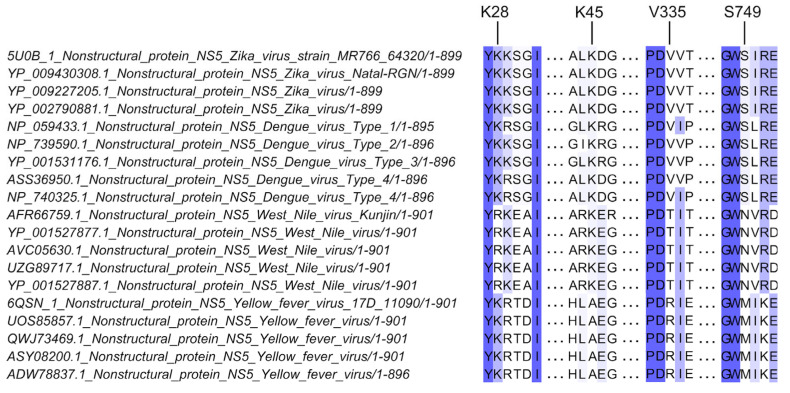
A representative sequence alignment of conserved surface residues in NS5 proteins of multiple mosquito-borne pathogenic flaviviruses. This alignment is modified from Dar et al., 2017 [19], to only select residues conserved between ZIKV and DENV—which are known to have direct interaction with human STAT2—but not conserved in West Nile virus (WNV) or Yellow fever virus (YFV), which do not directly interact with human STAT2. The purple highlights of residues reflect their level and degree of conservation across the flavivirus strains; highly conserved residues are highlighted in dark purple while less conserved residues are lighter in color. Labelled residues (K28, K45, V335, and S749) are the NS5 residues most likely involved in the efficiency of ZIKV in antagonizing STAT2. The NS5 protein sequences were aligned using the MEGA7 built-in MUSCLE tool according to their genomic position [20,22]. The Jalview program (version 2.11.3.2) was used to visualize the alignment [21].

A sequence alignment of multiple flavivirus NS5 was previously reported [19]. From this, 11 sequences from DENV, ZIKV, WNV, and YFV were chosen to determine the residues conserved between ZIKV and DENV (which directly interact with and antagonize STAT2), but not in WNV or YFV (which do not interact directly with STAT2). Four residues—K28, K45, V335, and S749—were identified and selected for investigation in this study (see Figure 1). Briefly, the NS5 protein sequences were aligned using the MEGA7 built-in MUSCLE tool according to their genomic position [20]. The Jalview program (version 2.11.3.2) was used to visualize the alignment [21].

### 2.2. Cells

African Green kidney cell line clones, Vero76 (ATCC CRL-1587^™^); adenocarcinoma human alveolar basal epithelial cells, A549 (ATCC CCL-185^™^); and its derivatives expressing bovine viral diarrhea virus, Npro protein (BVDV-Npro A549) and interferon α/β receptor knockout (IFNAR^−/−^) A549 cells, were cultured in DMEM (Gibco, Waltham, MA, USA). All culture media were fortified with 10% (*v/v*) fetal calf serum (FCS), 2 mM L-glutamine, 100 Units/mL penicillin, 100 µg/mL streptomycin. All mammalian cells were incubated at 37 °C in a 5% CO_2_ humified environment. BVDV-Npro A549 cells were kindly provided by R.E. Randall (University of St. Andrews, St. Andrews, UK); IFNAR^−/−^ A549 cells were generated using lentiCRISPRv2 protocol as described below. Modified cell lines were maintained with the addition of Blasticidin (10 µg/mL, Invivogen, Waltham, MA, USA) for BVDV-Npro A549, and Puromycin (1 µg/mL, Invivogen) for IFNAR^−/−^ A549 cells. All cell lines were regularly tested for the presence of mycoplasma and are free from mycoplasma contamination in this study.

### 2.3. Generation of Interferon α/β Receptor Knockout A549 Cells

Clustered regularly interspaced short palindromic repeats (CRISPR), with CRISPR-associated protein 9 (Cas9) technology, was used to silence and disable transcription of the IFNAR gene, also known as IFNAR knockout. This was done via a lentiviral CRISPR/Cas9 plasmid system targeting exons of the IFNAR genomic locus in A549 cells. The generation of the lentiviral CRISPR plasmid was performed according to the lentiCRISPRv2 protocol described on Dr. Feng Zhang’s website (https://zlab.bio/resources-2; http://sanjanalab.org/library/protocol_lentiOligo.pdf, both URLs accessed on 5 July 2019) [23,24]. The small guide RNA oligos (sgRNAs) were designed using a webtool CHOPCHOP (https://chopchop.cbu.uib.no/, accessed on 5 July 2019). After inputting human IFNAR1 as the target gene for editing, the resulting top two ranking sgRNAs were chosen: IFNAR#1 and IFNAR#2 (https://chopchop.cbu.uib.no/results/1711375478298.4287/details/1 and https://chopchop.cbu.uib.no/results/1711375478298.4287/details/2, accessed on 5 July 2019). These two sgRNAs targeted two specific exon regions within the IFNAR gene (IFNAR#1, 5′ TTCCATCAGATGCTTGTACGCGG 3′; IFNAR#2 5′ GACCCTAGTGCTCGTCGCCGTGG 3′).

HEK293T cells were first seeded at 4 × 10^5^ cells per well in six-well plates and transfected with a shuttle vector lentiCRISPRv2 containing human IFNAR small guide RNA (sgRNA) (Addgene #52961), envelope and packaging plasmids pVSVg (Addgene #8454), and psPAX2 (Addgene #12260) [23,24]. The following morning, cell media were replaced with 2 mL 10% DMEM. At 48 h, supernatants containing lentivirus particles were harvested and clarified using 0.45 μm filters to remove cellular debris before being aliquoted and stored at −80 °C for further use.

To facilitate knockout of IFNAR via CRISPR/Cas9 in A549 cells by lentiviral transduction, 500 µL filtered lentivirus supernatant(s) was added into wells of A549 cells seeded in six-well plates at 75% confluency and incubated for 48 h. After this, transduced cells were expanded into T75 tissue culture flasks, and culture media were replaced with 10 mL 10% DMEM containing 1 µg/mL puromycin and incubated for 72 h. IFNAR knockout (IFNAR^−/−^)-transduced A549 cells were periodically compared with cells transduced with an enhanced green fluorescent protein (eGFP) expression construct (positive control) and an empty expression construct (negative control) to determine the efficiency of transduction and the time for population expansion. Transduced cells were then expanded into T175 tissue culture flasks and cultured under puromycin conditions for 2 further passages. The IFNAR knockout efficiency in IFNAR^−/−^ A549 cells after 3 passages of puromycin selection was evaluated by Illumina sequencing and Western blotting.

### 2.4. Western Blotting

Cells were seeded at 8 × 10^5^ cells per well in six-well plates for 12 h and incubated at 37 °C in 5% CO_2_. The following day, cell monolayers were lysed in ice-cold NP40 lysis buffer [50 mM Tris pH 8.0, 150 mM NaCl, 1% (*v/v*) Nonidet P40 (NP40), protease inhibitor cocktail (Sigma Merck, Burlington, MA, USA)], collected into microfuge tubes, and stored at −80 °C until further use. The protocol was then performed as previously described [25]. After the blocking step, membranes were incubated overnight with respective concentrations of primary antibodies—0.1 μg/mL rabbit anti-pSTAT1 (Cell Signaling Technology, Danvers, MA, USA, [CST], #9167), 1:2000 diluted rabbit anti-STAT2 (CST, #72604), 1:2000 diluted rabbit anti-ZIKV-NS5 (kind gift from Prof. Andrés Merits, University of Tartú, Estonia), and 1:20,000 diluted mouse anti-GAPDH (Sigma, St. Louis, MO, USA, G8795)—in 1× TBS plus 0.1% Tween 20 (TBS-T) blocking solution at 4 °C. The following day, membranes were washed 6× with TBS-T and probed with corresponding secondary goat anti-rabbit and anti-mouse HRP conjugate antibodies (Cell Signaling Technology, #7074 and #7076) diluted at 1:20,000 in blocking solution at rt for 1 h. Lastly, membranes were washed 4× with TBS-T and twice with 1× TBS prior to enhanced chemiluminescence (ECL) development using SuperSignal^™^ West Pico PLUS chemiluminescent substrate (Thermo Scientific, Waltham, MA, USA). Proteins of interest were detected and visualized on the Amersham^™^ Imager 600 system (GE Life Sciences, Marlborough, MA, USA) with auto exposure settings. The fold-enrichment of the relative STAT2 per NS5 protein level was determined by densitometric quantitation using ImageJ (v1.53t) software.

### 2.5. Generation of Viruses with Substitutions at Selected NS5 Residues by Circular Polymerase Extension Reaction

The respective ZIKV-NS5 mutants (K28A/R, K45, V335A/T, S749A/N) were generated based on ZIKV-Natal isolate using circular polymerase extension reaction (CPER) as previously described [26,27,28]. Using mutagenesis primer pairs ordered from Integrated DNA Technologies (IDT, SG) (Table S1), mutations K28A (AAA→GCA; Lysine[K]→Alanine[A]), K28R (AAA→AGA; Lysine[K]→Arginine[R]), K45A (AAG→GCG; Lysine[K]→Alanine[A]), V335A (GTG→GCG; Valine[V]→Alanine[A]), V335T (GTG→ACG; Valine[V]→Threonine[T]), S749A (AGC→GCC; Serine[S]→Alanine[A]), and S749N (AGC→AAC; Serine[S]→Asparagine[N]) were introduced into ZIKV-Natal CPER fragments 6 or 7. All substitutions were introduced in two overlapping PCR fragments containing the same substitution site at the overlapping region. For the K28A/R and K45A substitutions, ZIKV-Natal CPER fragment 5 was used instead of fragment 7 as an overlapping fragment to pair with fragment 6 (Table S1). Respective mutagenized fragments 5, 6, and 7 were gel-purified and verified by Sanger sequencing to ensure the respective substitutions were retained. Seven separate CPERs were performed using PrimeSTAR GXL DNA polymerase (TaKaRa, San Jose, CA, USA) with equimolar amounts (0.1 pmol each) of ZIKV-Natal CPER fragment and respective fragments containing substitutions, as previously described [27]. The completed CPER products were transfected onto Vero76 cells to recover the respective viruses with substitutions in NS5. These NS5 variants were further propagated in C6/36 cells at a multiplicity of infection (MOI) of 0.01 to generate passage 1 (p1) working stocks for experiments. The retention of mutations in all viral working stocks was verified by Sanger sequencing.

### 2.6. Growth Kinetics

For the viral growth kinetics assay, cells were seeded at 4 × 10^5^ cells per well in six-well plates 12 h prior to the experiment. Cells were then infected with passage 1 stocks of respective viruses at the indicated multiplicity of infection (MOI) in the figures. At the timepoints after infection indicated in the figures, 120 µL of culture supernatant was collected from each sample well and stored at −80 °C until sample titration by immuno-plaque assay on Vero76 cells, as previously described [29]. Three independent experiments were performed for each cell line.

### 2.7. Immunofluorescence

Indirect immunofluorescence assay (IFA) was performed to detect ZIKV-NS5 protein in the nuclei of Vero76 and WT-A549 cells infected with K28A, K28R mutants, and wildtype ZIKV-Natal. Briefly, 1 × 10^5^ cells per well were seeded on coverslips in 24-well plates 12 h prior to being infected with respective strains of ZIKV at MOI of 1.0. Monolayers were fixed in 100% acetone at −20 °C for 5 min. Then, the coverslips were transferred onto a fresh 24-well plate and washed thrice with 1× PBS with 0.05% Tween20 (0.05% PBS-T). Then, the cells were incubated in 1× Pierce^™^ Clear Milk blocking buffer in 1× PBS solution at rt for 1 h. Following the blocking step, the blocking solution was removed, and cells were incubated in 1:200 diluted ZIKV-specific anti-NS5 rabbit polyclonal antibody at rt for 1 h. Next, cells were washed thrice with 0.05% 1× PBS-T, and then incubated in 1:1000 diluted anti-rabbit Alexa^®^ Fluor-488 (Invitrogen, #A-11034) at rt for 30 min. Lastly, cells were washed thrice with 0.05% 1× PBS-T and mounted onto glass slides using Prolong™ Diamond antifade mounting solution with DAPI (Life Technologies, Carlsbad, CA, USA). Fluorescence images were visualized at 63× objective magnification (630× final magnification; 63× objective; and 10× ocular) using the Carl Zeiss LSM900 upright confocal microscope (Carl Zeiss, Oberkochen, Germany). Scale bars on the bottom right corner of all merged images denote 10 µm. Two independent experiments were performed.

### 2.8. Statistical Analyses

The data were analyzed using GraphPad Prism 9.0.1 software (La Jolla, CA, USA). Two-way analysis of variance (ANOVA) with Tukey’s post-hoc multiple comparisons test was used to compare within groups and discern the differences between the groups, unless otherwise specified in the figure legends. Statistical differences for all experiments were set at *p* value < 0.05, unless otherwise specified.

## 3. Results

### 3.1. Substitutions at Residues K28, K45, V335, and S749 Attenuated Virus Replication in Type I IFN Competent Cells

To narrow down the residues of interest to be experimentally validated, further analysis was performed based on a sequence alignment of multiple flavivirus NS5 sequences to identify key NS5 residues that are likely to be involved in direct interaction with STAT2. Residues that are conserved between ZIKV and DENV (which directly interact with STAT2), but not in WNV or YFV (which do not interact directly with STAT2), were chosen as key NS5 residues likely to be involved in STAT2 antagonism. Of these, four NS5 residues—K28, K45, V335, and S749—were selected for further characterization (Figure 1). Although residues K28 and K45 were not uniformly conserved between WNV and YFV, residues K28 and K45 were still included for further characterization as both residues are involved in interactions with human STAT2, as well as in other NS5 functions [19,30,31].

**Figure 2:**
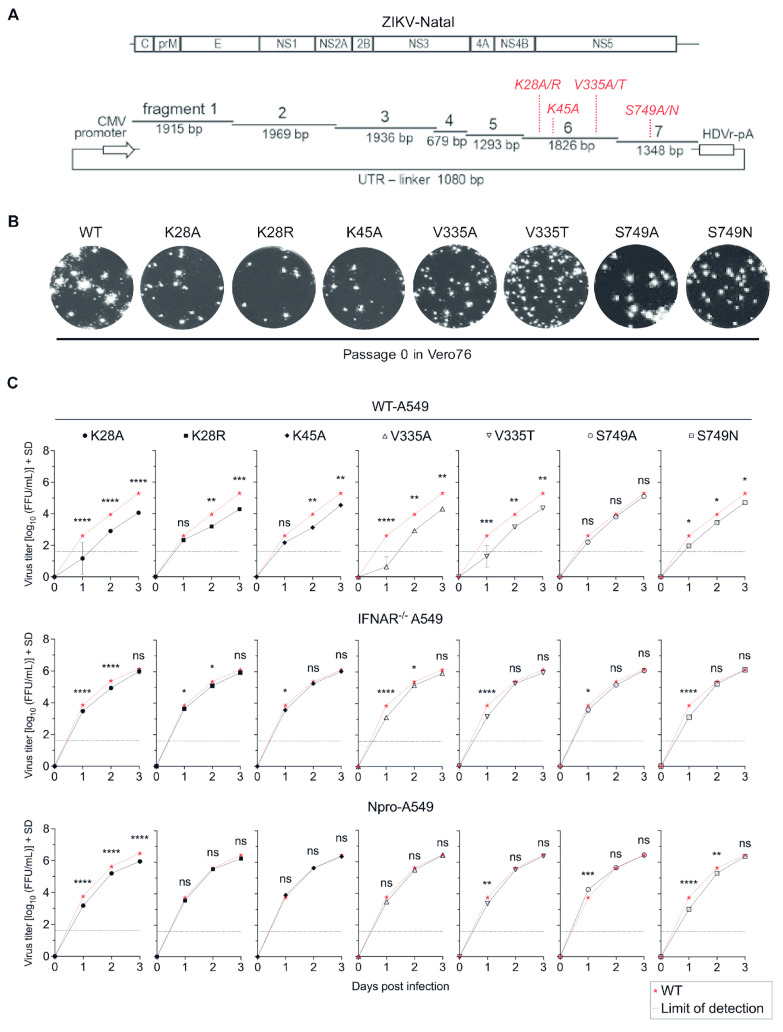
Generation and characterization of ZIKV with substitutions in NS5 by CPER. (**A**) Substitutions are introduced as two overlapping fragments (both containing the respective substitutions in the overlapping region), which then replace Natal-CPER fragments 5 and 6 or 6 and 7 during CPER. (**B**) Foci of respective NS5 alignment ZIKV mutants recovered from transfection of CPER product in Vero76 cells, as determined by titrating harvested supernatants containing respective mutant viruses using immuno-plaque assay (iPA). (**C**) Growth kinetics of viruses with substitutions in NS5 against wildtype ZIKV-Natal in WT-A549, IFNAR^−/−^ A549, and BVDV-Npro A549 cells. Shown are the titer values in log_10_ after infection with respective ZIKV strains at MOI 0.1, determined by foci-forming units (FFUs)/mL on Vero76 cells. Error bars show mean ± standard deviation (SD); n = 3 independent experiments. Dotted line represents detection limit. Pairwise comparisons of the means of virus titers between WT virus and selected mutants (as indicated) were performed and statistical differences were determined using two-way ANOVA with Tukey’s post-hoc test, where *p* < 0.05 (95.0% confidence); “ns”, not significant, * *p* < 0.05, ** *p* < 0.005, *** *p* < 0.0005, **** *p* < 0.0001. Limit of detection for iPA is 1.6 log_10_FFU /mL or 40 FFU/mL.

Substitutions of alanine (A) and residues conserved in non-STAT2-interacting flaviviruses (WNV/YFV) at the four selected positions (Figure 2A) were introduced into corresponding fragments 6 and 7 of the ZIKV-Natal CPER by overlapping PCR mutagenesis to generate seven viruses: K28A, K28R, K45A, V335A, V335T, S749A, and S749N. All substitutions were introduced as two overlapping DNA fragments containing the mutation site at the overlapping region. Seven CPERs were performed to generate passage 0 (p0) stocks of the respective mutant viruses from Vero76 cells (Figure 2B). Passage 1 (p1) viral working stocks were then propagated in C6/36 cells using passage 0 (p0) virus stocks for experiment uses. The presence of mutations in all mutant viruses was confirmed by Sanger sequencing.

To test if these substitutions are phenotypically relevant to the viral replication and/or modulation of IFN-signaling, the growth kinetics of the NS5 variants and the wildtype (WT) ZIKV-Natal were examined in WT-A549 cells (IFN-I response-competent), IFNAR^−/−^ A549, and BVDV-Npro A549 cells (both are IFN-I response impaired; IFN-I denotes type I IFN) at a multiplicity of infection (MOI) of 0.1 over a 3-day period. This is because IFN-I is the main antiviral response and the modulator of ZIKV replication in A549 cells, with IFN-III playing a less prominent role [32,33,34].

Most mutant viruses, except the S749A virus, replicated less efficiently compared to WT ZIKV-Natal in IFN-I response-competent WT-A549 cells (Figure 2C). The most significant effects on virus growth were consistently observed for substitutions at K28A (*p* ≤ 0.0001), V335A (*p* = 0.0073), and V335T (*p* = 0.0081) at 3 days post-infection (dpi). Interestingly, K28A substitution also attenuated virus replication in IFN-I response-impaired IFNAR^−/−^ and BVDV-Npro A549 cells, although virus titers for K28A virus were similar to those of the WT virus in IFNAR^−/−^ A549 cells at 3 dpi (Figure 2C). Additionally, less efficient virus replication was exhibited at early-to-mid timepoints (1 to 2 dpi) for V335A (*p* ≤ 0.0001; *p* = 0.0458) virus in IFNAR^−/−^ A549 cells, as well as for S749N virus (*p* ≤ 0.0001; *p* = 0.0022) in BVDV-Npro A549 cells (Figure 2C). Altogether, these findings indicate that mutations of the residues of interest selected from NS5 sequence alignment weaken in vitro ZIKV growth in IFN-I response-competent environments.

### 3.2. Substitutions at Selected Residues in NS5 Reduced ZIKV Efficiency to Antagonise STAT2

Since most of the examined NS5 substitutions attenuated ZIKV growth in vitro in IFN-I response-competent environments, we next proceeded to determine whether these substitutions could potentially affect the efficiency of ZIKV-NS5 in antagonizing STAT2. To do so, total STAT2 and ZIKV-NS5 levels from whole cell lysates of WT-A549 and IFNAR^−/−^ A549 cells infected with either mutant or wildtype ZIKV-Natal (WT) at MOI 5.0 were examined and compared using immunoblotting. A relatively high MOI of 5.0 was used to ensure that all cells were infected.

**Figure 3:**
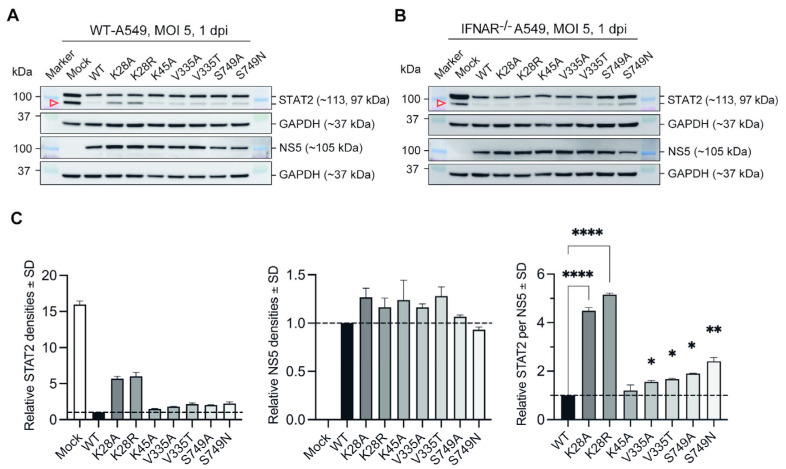
Substitutions at selected NS5 residues reduced the efficiency of ZIKV to antagonise STAT2. (**A**,**B**) Representative STAT2 and NS5 immunoblots of WT-A549 (**A**) and IFNAR^−/−^ A549 (**B**) cell lysates infected at MOI 5.0 with respective viruses or wildtype ZIKV-Natal (WT). Infected cell lysates were harvested at 1 dpi. Uninfected cells were included to differentiate the STAT2 levels under homeostatic cellular conditions. Respective blots were probed with either a primary antibody of rabbit anti-human STAT2 (CST, 1:2000), rabbit anti-ZIKV-NS5 protein (1:2000), or mouse anti-GAPDH (Sigma, 1:20,000). After washes with 0.1% 1xTBS-T, the blots were then probed with either a secondary anti-rabbit or anti-mouse HRP-linked conjugate antibody (CST, 1:5000). Two independent experiments were performed. Bands of respective proteins of interest, STAT2 (113, 97 kDa), NS5 (105 kDa), and GAPDH (37 kDa), were observed. (**C**) Densitometry analysis of STAT2 and NS5 immunoblots of WT-A549 cells infected with NS5 mutants or WT virus. The bands used for densitometry analysis are highlighted by a red triangle in the blots. Respective STAT2 and NS5 band densities were first divided, each with corresponding GAPDH band densities, to obtain normalized relative STAT2 and NS5 density values for each mutant or WT virus. The ratio of the total STAT2 levels per NS5 protein level from each sample were then determined by dividing the normalized STAT2 densities with normalized NS5 protein densities. Error bars show mean ± standard deviation (SD). Dotted lines represent the ratio of STAT2 to NS5 protein levels of WT virus. Statistically significant differences are shown for K28A, K28R, V335A, V335T, S749A, and S749N viruses against WT ZIKV-Natal. Statistical significances were determined using one-way ANOVA with Tukey’s post-hoc test, where *p* < 0.05 (95.0% confidence); * *p* < 0.05, ** *p* < 0.005, **** *p* < 0.0001.

There were two bands of STAT2 at 113 kDa (STAT2-1) and 97 kDa (STAT2-2) observed in the A549 cells in our assay. STAT2-2 is likely a truncated form of STAT2 due to alternative splicing from competing acceptor splice sites (P52630-4) [35,36,37], and is considered biochemically relevant as it is also observed in other cell lines [38,39]. Levels of both STAT2-1 and STAT2-2 were highest in uninfected WT-A549 and IFNAR^−/−^ A549 cells. In contrast, cells infected with ZIKV, irrespective of WT or mutant viruses, showed less STAT2-1 and STAT2-2 compared to uninfected WT-A549 cells (Figure 3A). Between the cells infected with WT and NS5 mutant viruses in WT-A549 cells, there were no observable differences to the STAT2-1 band.

However, differences were observed for the STAT2-2 band between the WT and NS5 mutant viruses (indicated by a red triangle), as all NS5 mutants showed a reduced ability to degrade STAT2-2 compared to the WT virus (Figure 3A). In particular, the K28A and K28R mutants appear to have completely lost their ability to degrade STAT2-2 (Figure 3A).

In IFNAR^−/−^ A549 cells, the levels of STAT2-1 were reflected similarly to those in WT-A549 cells. The levels of STAT2-1 and STAT2-2 were highest in uninfected cells, while STAT2-1 levels were similar between cells infected with WT and NS5 mutant viruses (Figure 3B). The levels of STAT2-2 (highlighted by a red triangle) were also similar between all NS5 mutant viruses and WT, with slightly more STAT2 (both isoforms) observed in cells infected with S749N mutant (Figure 3B). Likewise, NS5 protein levels were consistent between all mutants and the WT virus-infected IFNAR^−/−^ A549 cells (Figure 3B).

Densities of STAT2-2 and NS5 bands in immunoblots from WT-A549 cell lysates were measured and normalized with their corresponding GAPDH band densities to determine the relative STAT2 per NS5 protein levels between WT and NS5 mutant viruses. The normalized density values indicated that relative STAT2 levels were higher in cells infected with all NS5 mutants compared to WT virus-infected cells (Figure 3C). The ratios of STAT2 per NS5 protein were the highest in K28A and K28R infected cells, at 4.49 ± 0.09 and 5.15 ± 0.04-fold higher than in WT, respectively, while other mutants showed more modest increases (Figure 3C). Altogether, these findings demonstrate that mutations of selected residues of interest from NS5 alignment attenuate the efficiency of ZIKV-NS5 to antagonize STAT2, most likely via proteasomal degradation, especially for residue K28.

### 3.3. Substitutions at Residue K28 Resulted in Different NS5 Nuclear Body Phenotypes

**Figure 4:**
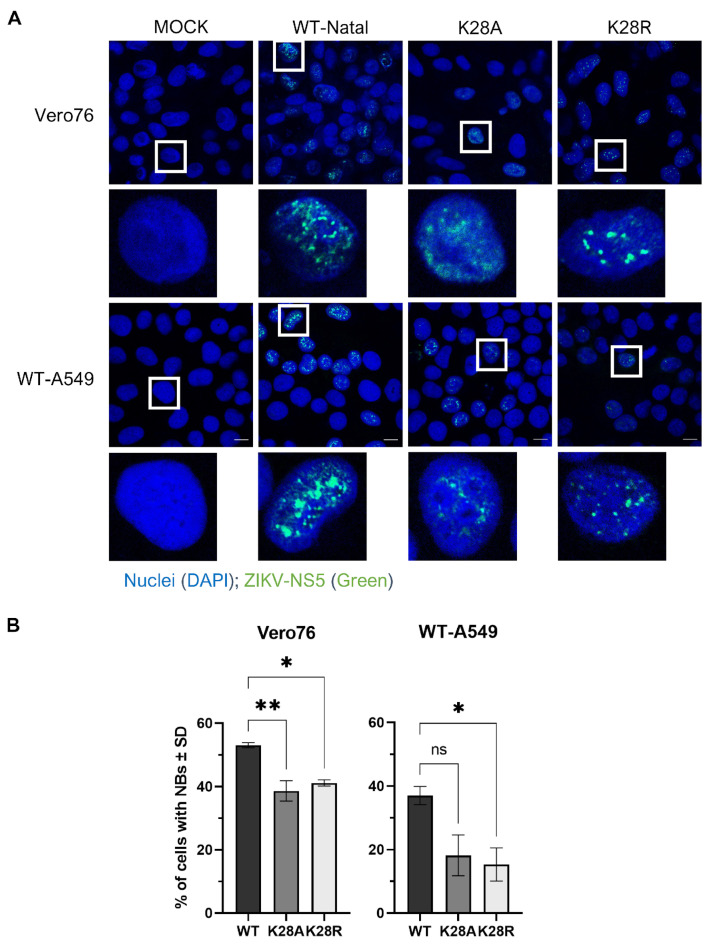
Substitutions at residue K28 induce different NS5 nuclear body phenotypes in Vero76 and WT-A549 cells. (**A**) Representative immunofluorescence images of ZIKV-NS5 expression in ZIKV-infected cells at 1 day p.i. Respective cells were infected with ZIKV at MOI 1.0. Levels of ZIKV-NS5 in cells were probed using a rabbit anti-ZIKV-NS5 primary antibody and an Alexa^®^ Fluor-488 anti-rabbit conjugate secondary antibody. Nuclei were stained with DAPI. Fluorescence-labelled cells were visualized at 630× final magnification (63× objective and 10× ocular). Scale bars on the bottom right corner of all merged images denote 10 µm. Two independent experiments were performed. (**B**) A bar graph representing the quantification of the number of ZIKV-NS5-positive cells with NBs for WT, K28A, and K28R virus infections in two independent experiments. Error bars show mean ± standard deviation (SD). Statistical significances were determined using one-way ANOVA with Tukey’s post-hoc test, where *p* < 0.05 (95.0% confidence); “ns”, not significant, * *p* < 0.05, and ** *p* < 0.005.

Thus far, the experiments performed demonstrated that substitutions at residue K28 (K28A/R) exhibited the strongest effect on ZIKV replication (Figure 2) and its antagonism of STAT2 (Figure 3) compared to substitutions at other residues of interest (K45, V335, and S749). Like for DENV, the localization of NS5 to the nucleus and the formation of distinct NS5 nuclear bodies (NBs) were observed for ZIKV [40,41]. However, only the impact of ZIKV-NS5 nuclear localization on the inhibition of IFN expression in cells has been shown [40,42]. Furthermore, DENV and ZIKV NS5 form NBs with different morphologies [43,44], suggesting that these different morphologies of ZIKV-NS5 NBs may have a functional importance. As residue K28 is located in a non-canonical nuclear localization signal (NLS) region (nucleotides 11 to 90) [42], we examined whether mutations K28A/R could affect nuclear translocation and the formation and shape of NS5 NBs. To do this, Vero76 and WT-A549 cells were infected with either K28A, K28R, or WT viruses at MOI 1.0, and the ZIKV-NS5 nuclear localizations and phenotypes of NBs were visualized using immunofluorescence with anti-NS5 antibodies at 1 day post-infection (dpi), as previously described [40]. Substitutions at K28A and K28R displayed different phenotypes of NS5-containing NBs in WT-A549 and Vero76 cells, with the differences more pronounced in Vero76 cells (Figure 4).

Furthermore, K28A and K28R viruses each displayed different NB phenotypes from each other, with K28A displaying diffused and small punctuated NBs while K28R displayed larger and more distinct spherical NBs, more similar to those observed in WT virus infection (Figure 4A). As a measure of whether different NB morphologies were likely to be represented consistently among infected cells, the numbers of infected cells containing characteristics of each NB phenotype were quantified. There were more NBs present in cells infected with WT virus compared to those infected with K28A and K28R mutant viruses (Figure 4B). In essence, these findings indicate that substitutions at residue K28 alter the appearance of the NS5 NBs, with the K28A mutation exhibiting the stronger effect.

## 4. Discussion

Among clinically important flaviviruses such as DENV, ZIKV, WNV, and YFV, only DENV and ZIKV antagonize STAT2 signaling activity by facilitating proteasomal degradation of STAT2 via direct NS5-STAT2 interaction [1,3,6,7,16]. Furthermore, ZIKV can also antagonize STAT2 activity by directly interacting with STAT2 to prevent its binding to IRF9 and the formation of the ISGF3 complex [17].

Previous studies have predicted and validated several ZIKV-NS5 surface residues that physically interact with human STAT2 to facilitate the antagonism of STAT2 [17,19]. However, not all the reported NS5 residues were experimentally validated at the time of this study’s commencement. The NS5 residues that were experimentally validated were Y25, R327, G338, D734, and H855 [17]. Here, a sequence alignment of multiple flavivirus NS5 proteins was first conducted to narrow down candidate residues in ZIKV-NS5 protein for further evaluation of their potential functions in virus replication and STAT2 interaction/antagonism.

Using an alignment of multiple flavivirus NS5 sequences [41], the sequences of direct STAT2-interacting flaviviruses (ZIKV and DENV) were compared against non STAT2-interacting flaviviruses (WNV and/or YFV) to identify residues that shared the same amino acid preferences between ZIKV and DENV, but not in WNV and/or YFV. Four residues (K28, K45, V335, and S749) were selected as potential STAT2 interacting residues for validation. Substitutions of alanine (A) and of the amino acids (aa) conserved in non STAT2-interacting flaviviruses (WNV and/or YFV) were engineered into these selected residues to generate seven single mutant viruses using the CPER mutagenesis approach: K28A, K28R, K45A, V335A, V335T, S749A, and S749N.

To verify whether the four selected NS5 residues are associated with virus replication and ZIKV’s efficiency in antagonizing STAT2, substitutions at these positions were evaluated by examining the virus growth kinetics and the total STAT2 levels in ZIKV-infected cells with competent and deficient IFN-I responses. The results showed that all substitutions attenuated ZIKV replication in vitro in an IFN-I response-competent environment and reduced the efficiency of ZIKV in antagonizing STAT2. Substitutions at residue K28 (K28A/R) exhibited the strongest effect on impeding virus replication in IFN-I response-competent cells and considerably reduced the virus’s efficiency in antagonizing STAT2, likely via proteasomal degradation, compared to the substitutions in other selected positions.

Residues K28, K45, V335, and S749 are surface residues predicted to have direct biophysical interactions with human STAT2 [19,41]. The cryo-EM structure of the NS5-STAT-2 complex identified several NS5 residues involved in interaction between NS5 and STAT2 to facilitate its antagonism [17]. Among residues identified using cryo-EM, V335 was determined to interact with residues Q169, D171, and V172 of the coiled-coil domain of human STAT2 via a relatively weak van der Waals force [17]. This might also explain why substitutions at residue V335 in our study exhibited modest effects on the efficiency of ZIKV in antagonising STAT2, since V335 is not a key STAT2-interacting residue, but rather a part of an interaction region—which includes two neighbouring surface residues, V336 and T337—that reinforces NS5-STAT2 binding.

It was surprising to find that K28 might play a more pronounced role in virus replication in IFN-I response-competent cells compared to the other selected residues. This could potentially be because residue K28 is also involved in guanylyltransferase (GTase) activity, as it is located in the guanosine-5′-triphosphate (GTP) binding site of the methyltransferase (MTase) domain, as observed in ZIKV and other flaviviruses such as Wesselsbron virus and WNV [30,45,46,47]. The GTP binding site is essential for the utilization of GTP as a substrate to catalyze both N7-guanosine and ribose 2′-oxygen (2′-O) methylation reactions that lead to the formation of a methylated 5′-end cap for nascent ZIKV RNA genome [48,49]. A 5′ methylated cap is necessary for the stability and translation of viral RNA, as well as for evading the activation of the host’s antiviral pathogen recognition receptors such as retinoic acid-inducible gene I (RIG-I) [50]. Therefore, mutations to residue K28 of ZIKV-NS5 may have more pronounced negative impacts on ZIKV growth by potentially inhibiting GTase activity, and thereby the catalyzation of the 5′-methylated cap, similar to observations of WNV and DENV [51,52].

Recently, residue K28 was also shown to be a key residue for MTase-MTase contact to mediate NS5 dimerization and dimer stabilization [31]. A crystallography study of ZIKV-NS5 oligomeric arrangements revealed that the dimerization of ZIKV-NS5 is facilitated by MTase-MTase and/or MTase-RdRp interactions via residues Y25, K28, and K29 [31]. The study also found that mutations to these residues impaired NS5 dimerization, which, in turn, negatively affected the GTase activity of MTase and the RNA synthesis efficiency of RdRp [31]. Furthermore, NS5 dimers are required for NS3 helicase to bind and facilitate the unwinding of viral dsRNA intermediate, and to remove the 5′ γ-phosphate from nascent viral RNA prior to the addition of 5′ methylated cap [53,54,55]. Hence, an impairment to NS5 dimer formation would mutually down-modulate the helicase activity of NS3, which would then negatively disrupt the addition of 5′ methylated cap to nascent viral genome. Future studies could be performed to determine the potential effects of K28A substitution on NS3 helicase activity and NS5 GTPase activity, as well as NS5 binding to STAT2 using expression constructs and appropriate assays to further characterize the role of this mutation in corresponding NS5 functions.

Furthermore, ZIKV-NS5 dimers were also demonstrated to be a key mechanism facilitating ZIKV neuropathogenesis, and that residue K28, along with three other residues, were key mediators for this [56]. Thus, the findings presented in this study on the negative impacts on ZIKV replication in vitro, its efficiency in antagonising STAT2, and its ability to form different NS5 nuclear body phenotypes might correlate with potential alterations to the dimerization ability of ZIKV-NS5 and 5′-methyl’s capping of nascent viral RNA, due to the substitutions at residue K28. Additionally, the impact on ZIKV-NS5 nuclear body phenotypes could also be indirectly associated with the disruption of the post-translational small ubiquitin-like modifier (SUMO)-ylation pathway. Given that SUMOylation is a post-translational modification of adding SUMO proteins to lysine residues of nuclei proteins to regulate the nucleoplasm activity of the host cell, and that lysine (K) is the native residue at position 28 of ZIKV-NS5, there could be a non-canonical SUMOylation-associated impact caused by residue K28, given that ZIKV-NS5 was demonstrated to reduce STAT2 expression by dissociating SUMO-1 protein from its key interacting promyelocytic leukemia (PML) partner protein via the SUMO-interacting residue, K252 [57]. This could further explain why mutations to residue K28 elicit a stronger detrimental impact to ZIKV’s growth and its efficiency to antagonize STAT2 compared to the mutations in the other residues.

On the other hand, K45A mutant exhibits a similar growth curve to K28R mutant in WT-A549 cells, but did not attenuate the efficiency of ZIKV to antagonize STAT2 in WT-A549 cells achieved by substitutions at residue K28. We hypothesize that this is likely because substitutions at residue K45 likely only affected the polar and hydrophobic intermolecular contact necessary for NS5 dimerization via MTase-to-MTase contact [31]. Residue K45 is an α3-helix residue that interacts with the α2-β1 loop (which residue K28 is a part of) for MTase-MTase contact, but unlike residue K28, residue K45 is not a key residue mediating the dimerization of NS5 [31]. Additionally, residue K45 is not located in any other enzymatically active regions in the MTase domain, unlike residue K28 [30]. Therefore, given the hypothesized lesser role of residue K45 in NS5 activity, this explains why mutations to residue K45 only affect virus replication in terms of virus genome replication but not STAT2 degradation [31].

Crucially, although our data have demonstrated that mutations at residue K28 attenuated the efficiency of ZIKV to antagonize STAT2, it was only evaluated in an infection model comprising A549 cells with competent and defective IFN-I-signaling. Our study also could not perform super-high-resolution imaging, as used by Ng et al., to analyze the structural differences of the NBs between K28A and K28R substitutions or provide qualitative observations to correlate the differences in nuclear body phenotypes to the different amino acids substituted [40]. Additionally, no orthogonal experiments were performed to further demonstrate the impact of substitutions at residue K28 on STAT2 antagonism by ZIKV, or to comprehensively address the potential molecular mechanisms behind the impacts of substitutions at the four selected NS5 residues. Hence, to solidify the findings presented in this study, future experiments should further examine the impacts of substitutions at these four selected NS5 residues on ZIKV’s growth and ability to antagonize STAT2 in ZIKV-tropic cells, such as NPCs and placenta trophoblasts, to observe whether the impacts of any substitutions at those residues would affect the neuropathogenesis of ZIKV. Moreover, co-immunoprecipitation assays involving STAT2 and NS5 proteins containing those substitutions could be performed to evaluate the effects on NS5-STAT2 binding. Likewise, the impacts on the RdRp replicase function caused by substitutions at residue K28 could be evaluated using a reporter replicon expressing a luciferase reporter for RNA replication, or by quantifying and comparing ZIKV RNA copies from cells infected with K28A, K28R, or WT viruses. This would reinforce our findings regarding the impact on STAT2 antagonism by ZIKV caused by substitutions at residue K28. Lastly, immunostaining of SUMOylation pathway proteins such as SUMO-1 and PML could provide deeper insights behind the differences in NB phenotypes observed between K28A and K28R substitutions.

In summary, our study has identified and investigated four residues (K28, K54, V335, and S749) in ZIKV-NS5 with potential implication in virus replication and STAT2 antagonism. We demonstrated that mutations at residue K28 significantly reduce virus replication and the efficiency of ZIKV to antagonize STAT2 as well as affected phenotypes of NS5 nuclear bodies. Collectively, our findings demonstrate that residue K28, identified through sequence alignment, is likely an important determinant of virus replication and STAT2 antagonism by ZIKV. This adds residue K28 and residue K45 to the number of other previously identified viral determinants in ZIKV-NS5 involved in viral replication and/or antagonism of innate immune responses, as well as making them potential target sites for developing an antiviral mutagenic RdRp inhibitor against ZIKV [42,57,58].

## Data Availability

Data are contained within the article (and Supplementary Materials).

## Supplementary Materials

The following supporting information can be downloaded at: https://www.mdpi.com/article/10.3390/microorganisms12040660/s1, Table S1: List of primers used in this study.
