# Supplementary material for: Residue K28 of Zika Virus NS5 Protein Is Implicated in Virus Replication and Antagonism of STAT2
Source: Microorganisms. 2024 Mar 26;12(4):660. doi: 10.3390/microorganisms12040660 (PMC11052099; doi:10.3390/microorganisms12040660)
Supplement: Supplementary file 1 [file microorganisms-12-00660-s001.zip › microorganisms-2841936-supplementary.pdf]

Table S1. List of primers used in this study.

List of primers

Primers used to generate DNA fragments and UTR-linker for ZIKV-Natal CPER and Sanger sequencing (5′–3′)	
Natal-CPER_F1_for	AGTTGTTGATCTGTGTGAATCAGAC
Natal-CPER_F1_rev	GTGAACGCTGCGGTACACAAGGAGTATG
Natal-CPER_F2_for	CATACTCCTTGTGTACCGCAGCGTTCAC
Natal-CPER_F2_rev	CTTTCACGGGGTGTCCAATTAGCTCTGAAG
Natal-CPER_F3_for	CTTCAGAGCTAATTGGACACCCCGTGAAAG
Natal-CPER_F3_rev	GACCCGTTTTCCAGCCTTTGTCAGAC
Natal-CPER_F4_for	GTCTGACAAAGGCTGGAAAACGGGTC
Natal-CPER_F4_rev	GAACAAACTCTGGCGTCCATCCACCT
Natal-CPER_F5_for	AGGTGGATGGACGCCAGAGTTTGTTC
Natal-CPER_F5_rev	TCCTGTTCCACCCCCACGTCTCTT
Natal-CPER_F6_for	AAGAGACGTGGGGGTGGAACAGGA
Natal-CPER_F6_rev	ACTTGTCCGCTCCCCCTTTGGTCTT
Natal-CPER_F7_for	AAGACCAAAGGGGGAGCGGACAAGT
Natal-CPER_F7_rev	AGACCCATGGATTTCCCCACACCG
Natal-CPER_UTR-linker_for (CMV)	GGTGTGGGGAAATCCATGGGTCTGGGTCGGCATGGCATCTCCACC
Natal-CPER_UTR-linker_rev (CMV)	GTCTGATTCACACAGATCAACAACTCGGTTCACTAAACGAGCTCTGCTTATATAG

Mutagenesis primers to introduce mutations K28A/R, K45A, V335A/T and S749A/N		
K28A_for	GGAGTTCTACTCCTACGCAAAGTCAGGCATCACC	Pair with Natal-CPER_F6_rev primer. Product replaces Natal-CPER fragment 6.
K28A_rev	GGTGATGCCTGACTTTGCGTAGGAGTAGAACTCC	Pair with Natal-CPER_F5_for primer. Product replaces Natal-CPER fragment 5.
K28R_for	CCTCTCTAGTAAACGGGGTTGTCAGG	Pair with Natal-CPER_F6_rev primer. Product replaces Natal-CPER fragment 6.
K28R_rev	CCTGACAACCCCGTTTACTAGAGAGG	Pair with Natal-CPER_F5_for primer. Product replaces Natal-CPER fragment 5.
K45A_for	CCGCGCCCTCGCGGATGGTGTGGCAACGGGAG	Pair with Natal-CPER_F6_rev primer. Product replaces Natal-CPER fragment 6.
K45A_rev	CTCCCGTTGCCACACCATCCGCGAGGGCGCGG	Pair with Natal-CPER_F5_for primer. Product replaces Natal-CPER fragment 5.
V335A_for	CAAAACCCTGGGATGCGGTGACTGGAGTCAC	Pair with Natal-CPER_F6_rev primer. Product replaces Natal-CPER fragment 6.
V335A_rev	GTGACTCCAGTCACCGCATCCCAGGGTTTTG	Pair with Natal-CPER_F6_for primer. Product replaces Natal-CPER fragment 6.
V335T_for	CAAAACCCTGGGATACGGTGACTGGAGTCAC	Pair with Natal-CPER_F6_rev primer. Product replaces Natal-CPER fragment 6.
V335T_rev	GTGACTCCAGTCACCGTATCCCAGGGTTTTG	Pair with Natal-CPER_F6_for primer. Product replaces Natal-CPER fragment 6.
S749A_for	GGGCGGGATGGGCCATCCGGGAGACTG	Pair with Natal-CPER_F7_rev primer. Product replaces Natal-CPER fragment 7.
S749A_rev	CAGTCTCCCGGATGGCCCATCCCGCCC	Pair with Natal-CPER_F6_for primer. Product replaces Natal-CPER fragment 6.
S749N_for	GGGCGGGATGGAACATCCGGGAGACTG	Pair with Natal-CPER_F7_rev primer. Product replaces Natal-CPER fragment 7.
S749N_rev	CAGTCTCCCGGATGTTCCATCCCGCCC	Pair with Natal-CPER_F6_for primer. Product replaces Natal-CPER fragment 6.

Nucleotides that are underlined represents the substitutions introduced
